# Dataset of output impedances of current sources by analytical method and by experimental method

**DOI:** 10.1016/j.dib.2018.11.050

**Published:** 2018-11-15

**Authors:** Bijay Kumar Sharma

**Affiliations:** a National Institute of Technology, Ashok Rajpath, Patna 800005, Bihar, India; b Indian Institute of Technology Patna, Bihta 801118, Bihar, India

## Abstract

The dataset provided in this article are related to the research article entitled “The Journey of Universal Hybrid-pi model-from its Inception to Experimental Validation and its impact on Analog Circuit Design” (Sharma, in press). While analyzing dataset of the incremental output impedances of the BJT Current Sources, Conventional Hybrid-pi model, and Unilateral Model grossly underestimate the output impedances whereas Universal Hybrid-pi Model gives a much larger range of output impedances from ro to 40ro. The quest for these enhanced prediction led to the discovery of “Variable Latching Effect” (Sharma, 1990). Furthermore the ascending order of the dataset of Break-over voltages of Device-under-Test (DUT) were obeyed by the dataset predictions of incremental output impedance by Universal Model but not obeyed by those made by Conventional Model and Unilateral model. Direct experimental measurement of output impedances of current sources using laboratory setup validated Universal Hybrid-pi Model (Sharma, 2003) [3] but the results were inconclusive. The experimental measurement of the incremental output impedances by a Professional setup was also done and verified by analytical results. All incremental analysis is carried out at a given Q-point and Q-point decides the incremental parameters of the Hybrid-pi model and T-model which are to be used in the analytic relations (2), (3) and (4) given in the main text (Sharma, in press). Q-points of the current sources at which the output impedance measurement have been made are given in this dataset (see Table 6). Model parameters at the given Q-points are derived from simple analytic relations given in the main text (Sharma, in press) and tabulated in Table 7 and Table 7A. The theoretical incremental output impedance are calculated for the conventional model, universal model and T-model and compared with the experimentally measured values of output impedance and tabulated in this dataset (see Table 8 and Figure 8). A very high gain Differential Amplifier׳s incremental voltage gain is experimentally measured and analytically verified. The experimental values and Universal Hybrid-pi model theoretical analytic results are given . The conventional model analytic results for incremental voltage gains are also tabulated. This article data is being made publicly available to enable critical or extended analysis.

**Specifications table**

**Table:** 

Subject area	*Physics*
More specific subject area	*Incremental Circuit Analysis, experimental measurement of output impedances ,impact induced instability device characterization*
Type of data	*Table, Graph,*
How data was acquired	*Theoretical Data was acquired through Incremental linear circuit analysis. Experimental Output impedance of the current source is acquired by measuring the reciprocal of the slope of Ic vs Vce curve. Slope is determined by taking the quotient of incremental current and incremental voltage. In dynamic measurement these increments are sinusoidal quantities with incremental amplitudes. In static testing the incremental values are DC increments. Break-over Voltages of the current sources using 2N2219A current sources were measured using Tek 370A curve tracer oscilloscope. Experimental values of output impedances of the eight current sources using 2N3055 are measured using Keithley’s Semiconductor Characterization System 4200.Analytic values are acquired using (2), (3) and (4) in the main text*[1]
Data format	*Analytical, experimental,*
Experimental factors	*Circuit instability when measuring Break-over voltages.*
Experimental features	*S-type Negative – Impedance Region (NIR) is observed in CE BJT (see*Fig. 3*in the main tex*t [1]*) with constant current drive at the base of Device-Under-Test(DUT) 2N2219A general purpose BJT. In the remaining current sources Circuit instability is marked by vertical I-V curves*
	*The laboratory set-up does the output impedance measurement under dynamic condition hence incremental voltages are sinusoidal and within the dynamic range so that the response is also pure sinusoidal. There should be no harmonic distortion.*
	*The professional set up does output impedance measurement under static conditions. Here incremental dc voltage is applied and incremental dc current is measured.*
	*Experimental set-up is shown in*Figs. 5*and*6*in the main text*[1].
	*The measurements have been kept within the dynamical range of the circuit.Static characterization while measuring the Q-point.*
	*Small signal condition maintained so that device(DUT) acts as a linear device.*
Data source location	*Patna, Bihar, India, Temperature 30°C, Latitude 25.5941°N and Longitude 85.1376°E.*
Data accessibility	*Data is in the main text article*[1]
Related Research Article	*Main Article*[1]*contains the related research work done by the author: the references are 1, 4, 27, 29, 30, 33, 34 given in the main article.*
	*Main Article*[1]*has related research work done by other scientists such as: Grens M.Sc. dissertation*[2], *Rein & Moller [17], Cresseler [18], Harame et.al. [19],Verzeldesi et.al. [21],Vendrame et.al. [22], Grans et.al. [25], Grinich & Jackson [31],Gray,Hurst,Lewis & Mayer [32] and Kraft et.al. [34].*

**Value of the data**

• Clearly Conventional Model and Unilateral model are inaccurate and misleading according to this dataset. Model-to hardware correlation demands the use of Universal Hybrid-pi model in future circuit analysis and design.
• In professional setup, the results are repeatable and reproducible and they can be used by scientific community.
• This experimental incremental voltage gain is verified by Universal Hybrid-pi model incremental circuit analysis hence Universal Hybrid-pi model gives the design rules for designing very high gain CE amplifiers.
• These design rules will particularly be useful in design of Op. Amp.

## 1 Data

In the present consolidated DIB Table 1, Tables 5 and 8 are being shared. In these Tables we have dataset of analytic result of output impedances by conventional hybrid-pi model, by unilateral model, and by Universal Hybrid-pi model. Here we also have dataset of Break-over voltages of the six current sources as measured by Tektronix 370 A curve tracer instrument.

**Table 1:** Dataset of analytic estimates of incremental output impedances of six current sources and their respective break-over voltages. DUT 2N2219A.

	C1	C2	C3	C4	C5	C6
R1	0.53r_0_	1r_0_	1.8r_0_	3.53r_0_	1.88r_0_	4r_0_
R2	1r_0_	1r_0_	2r_0_	4.12r_0_	1.97r_0_	4.19r_0_
R3	1.09r_0_	16.6r_0_	20.1r_0_	23.07r_0_	29.5r_0_	41r_0_
R4	60 V	108 V	112 V	116 V	118 V	128 V
R5	BV_CEO_	BV_CES_	BV_CEX1_	BV_CEX2_	BV_CEX3_	BV_CBO_

R1-Conventional Model analysis; R2-Unilateral Model analysis; R3-Universal Model analysis; R4-Experimentally observed Sustaining Voltage ; R5-Sustaining Voltage Symbol;

C1-CE BJT current source with constant base current drive;

C2- Current mirror current source;

C3-Symmetrical Widlar current source R_E1_= R_E1_=500 Ω;

C4-Symmetrical Widlar current source R_E1_= R_E1_=9 kΩ;

C5-Widlar current source R_E1_=0, R_E1_=500 Ω;

C6-Widlar current source R_E1_=0, R_E1_=9 kΩ.

In professional setup, data obtained are given in Tables 5 and 8.There are four dataset: Conventional model analytics dataset, Universal model analytics dataset, T_model analytics dataset, and experimentally determined static incremental output impedances of eight current sources.In professional setup, dataset of DC operating voltage and DC operating currents also known as Q-points of the current sources under consideration are given in Table 6. Incremental pi-parameters and T-model parameters are given Table 7. In differential amplifier incremental voltage gain experiment, 10 sets of measurements are made all within the dynamic range of the circuit so as to completely remove amplitude distortion and to obtain reliability of the measured values.These are given in Table 9.

While doing instability characterization, instability voltage BV_CEX_ (volts) is normalized in terms of r_0_=1/h_oe_=output impedance of CE BJT with constant current drive=I_C_/β_F_ at Base of DUT(device under test) by dividing BV_CEX_ (volts) by K=60 V/r_0_ .Table 2 gives the normalized instability voltages.

**Table 2:** Normalized instability voltages of 2N2219A NPN general purpose device with gold doping.

	C1	C2	C3	C4	C5	C6
BV_CEX_ (volts)	60	108	112	116	118	128
Normalized instability voltages(×r_0_)	1	1.8	1.866	1.933	1.966	2.133

C1-CE BJT current source with constant base current drive;

C2- Current mirror current source;

C3-Symmetrical Widlar current source R_E1_= R_E1_=500 Ω;

C4-Symmetrical Widlar current source R_E1_= R_E1_=9 kΩ;

C5-Widlar current source R_E1_=0, R_E1_=500 Ω;

C6-Widlar current source R_E1_=0, R_E1_=9 kΩ.

**Fig. 1:**
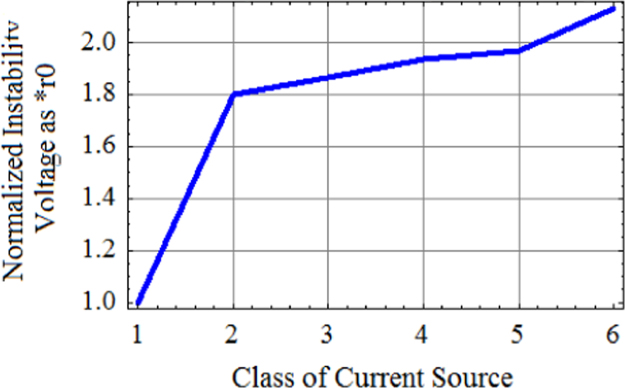
Normalized instability voltage points as multiples of r_0_ of progressively improved Class of current sources. Plot of analytic results from conventional Hybrid-pi model for six classes of current sources.

**Fig. 2:**
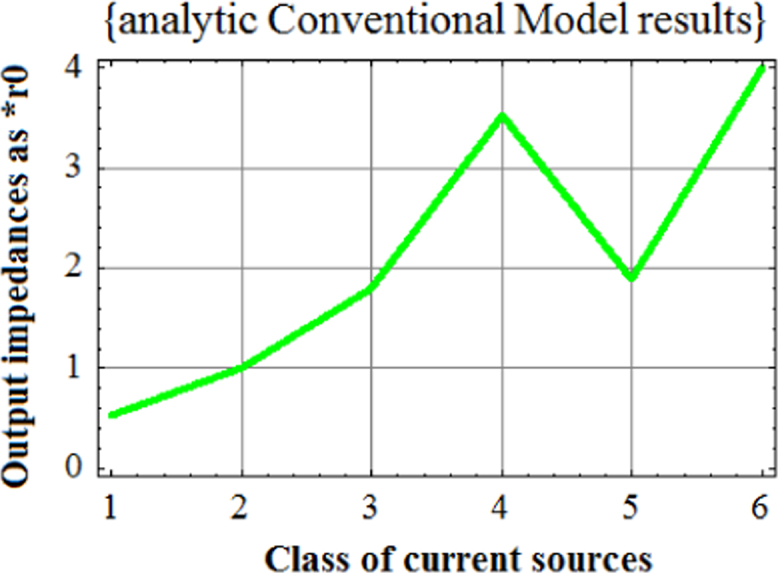
Plot of analytic results from conventional model for six classes of current sources. Plot of analytic results from Unilateral model for six classes of current sources.

**Fig. 3:**
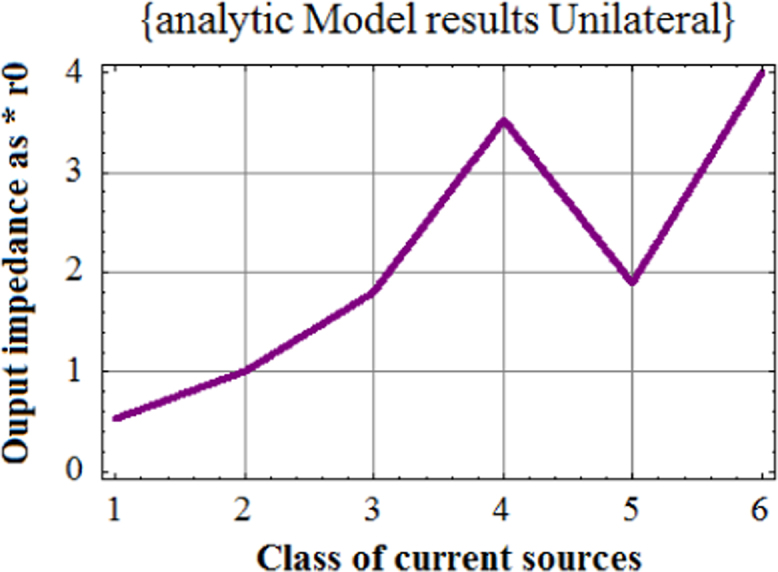
Plot of analytic results from unilateral model for six classes of current sources. Plot of analytic results from Universal Hybrid-pi model for six classes of current sources.

In all the figures the numbers on X-axis 1, 2, 3, 4, 5, and 6 correspond to Class VI, Class V, Class IV, Class III, Class II, and Class I current sources, respectively (Fig. 1, Fig. 2, Fig. 3).

Superposition of the plots of the four figures (that is the comparative study of normalized instability points and the analytic results of the output impedances from Conventional Hybrid-pi model, unilateral model, and from the Universal hybrid-model).

**Fig. 4:**
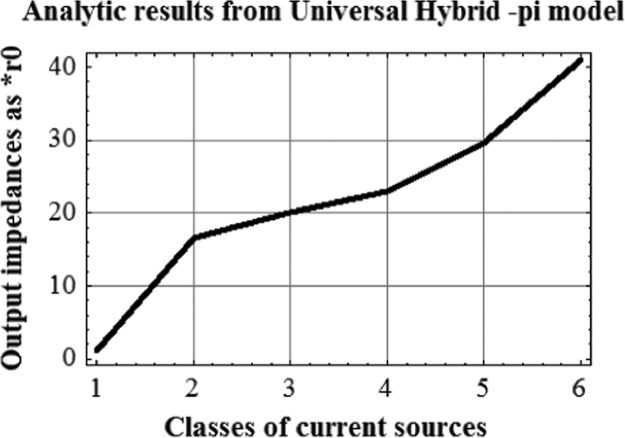
Plot of analytic results from Universal Hybrid-pi model for six classes of current sources. (This covers a much larger span of impedance. This triggered the discovery of VARIABLE LATCHING).

For complete superposition the graphs are made compatible by scaling down Fig. 4 by (1/8). The scaled down values are given in Table 3.

**Table 3:** Scaled down values of output impedances obtained from Universal hybrid-pi model. Scaling factor is (1/8).

	C1	C2	C3	C4	C5	C6
Output Impedance by Universal	1.09	16.6	20.1	23.07	29.5	41
1/8 Scaled down values (×r_0_)	0.136	2.075	2.5125	2.88375	3.6875	5.125

C1-CE BJT current source with constant base current drive;

C2- Current mirror current source;

C3-Symmetrical Widlar current source R_E1_= R_E1_=500 Ω;

C4-Symmetrical Widlar current source R_E1_= R_E1_=9 kΩ;

C5-Widlar current source R_E1_=0, R_E1_=500 Ω;

C6-Widlar current source R_E1_=0, R_E1_=9 kΩ.

**Fig. 5:**
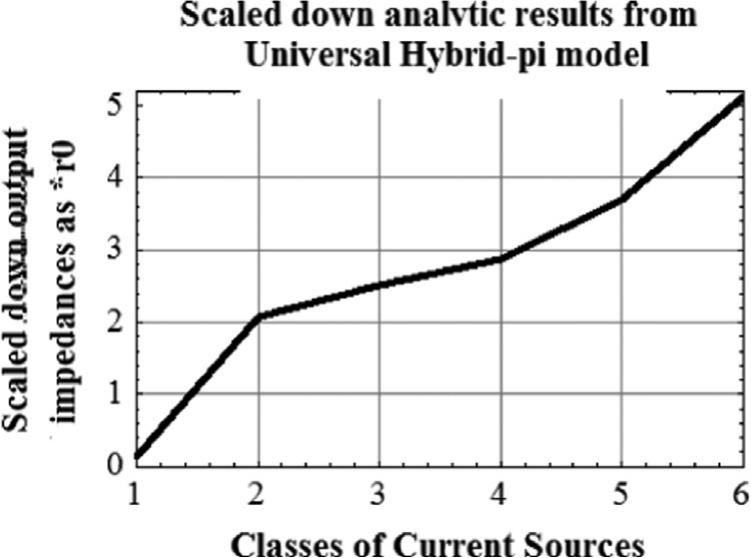
Plot of scaled down analytic results from Universal Hybrid-pi model for six classes of current sources. Scale factor=1/8.

After compatibility is achieved in the four plots (normalized instability plot, analytic results from conventional hybrid-pi model plot, analytic results from Unilateral model plot, and scaled down analytic results from Universal Hybrid-pi model plot), the upperposition is done by Show command (Fig. 5).

As seen from Fig. 4, analytic results from Universal Hybrid-pi Model give significantly enhanced output impedance of the six class of current sources as compared with those given in Fig. 2 (conventional) and Fig. 3 (unilateral) . It was this anomaly which triggered the finding of Variable Latching Effect in 1990.

**Fig. 6:**
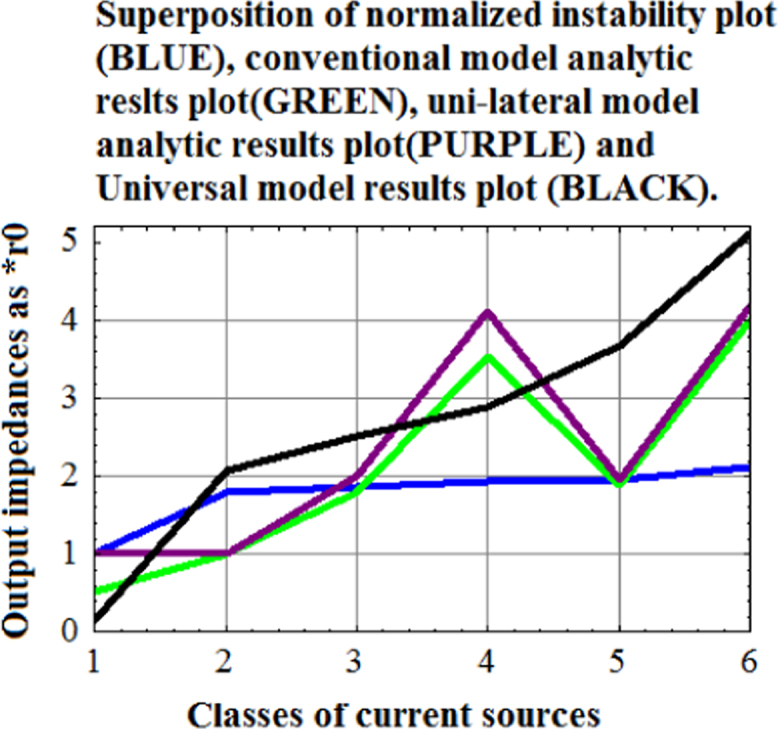
Superposition of normalized instability plot (BLUE), conventional analytic plot (GREEN), unilateral analytic plot (PURPLE) and scaled Universal-hybrid-pi plot (BLACK). (For interpretation of the references to color in this figure, the reader is referred to the web version of this article).

Fig. 6 shows that normalized instability plot (BLUE) obey the trend set by Universal-hybrid-pi model (BLACK).

Universal Model results (BLACK) follow the ascending trend of the normalized instability voltages of the current sources (BLUE). This correspondence indirectly validates Universal Hybrid-pi model as the correct small signal model of CE BJT at low frequencies.

Conventional model (GREEN) and unilateral model (PURPLE) have identical trends but quite contrary to the trend set by instability voltage (BLUE) and Universal hybrid-pi model(BLACK) (Table 4, Table 5, Table 6, Table 7, 7A,8,9).

**Table 4:** The output impedances achieved by experimental measurement in laboratory setup.

	C1	C2	C3	C4	C5
R1	61k	61.5k	61k±18k	123 V	BV_CEO_
R2	281k	300.5k	280k±36.25k	163 V	BV_CES_
R3	929k	974k	906.3k±90.4kk	180 V	BV_CEX1_
R4	1.03 M	1.09 M	906.3k±90.4k	188 V	BV_CEX2_
R5	1.47 M	1.5 M	906.3k±180.4k	192 V	BV_CEX3_

R1-CE BJT current source with constant base current drive given in Fig. 5 of the main text [1];

R2-Current mirror current source given in Fig. 6 of the main text[1];

R3-Symmetrical Widlar current source R_E1_=R_E1_=118.7 Ω given in Fig. 6 of the main text [1];

R4-Symmetrical Widlar current source R_E1_=R_E1_=148 Ω given in Fig. 6 of the main text [1];

R5-Symmetrical Widlar current source R_E1_=R_E1_=337 Ω given in Fig. 6 of the main text [1];

C1-Universal Model analysis; C2-T- Model analysis; C3-Experimentally measured Output impedance of five classes of current sources; C4-Experimentally observed sustaining voltage of the five class of current sources; C5-Sustaining voltage symbol.

**Table 5:** Professional setup output impedance of eight classes of current sources and by analysis based on Conventional Model, Universal Model, and T-Model.

**Class**	**R**_**0_CONV**_**(MΩ)**	**R**_**0_UNIV**_**(MΩ)**	**R**_**0_T-model**_**(MΩ)**	**R**_**0_Exp**_**(MΩ)**
**VIII**	**0.05**	**0.09**	**0.11**	**92k**
**VII**	**0.07**	**0.2**	**0.33**	N.A.^*^
**VI**	**0.28**	**0.8**	**1.24**	**747.8k**
**V**	**0.3**	**0.84**	**1.31**	**838.9k**
**IV**	**0.6**	**1.44**	**2.15**	**1.5M**
**III**	**0.37**	**1.06**	**1.74**	**1.127M**
**II**	**0.4**	**1.12**	**1.96**	**1.13M**
**I**	**0.8**	**2.23**	**3.53**	**2.23M**

^*^Class VII is current mirror. Without matching transistors, Current Mirror cannot be synthesized. Hence the result in this case has been rejected.

Current source 1 -Class VIII-CE BJT current source with constant base current drive given in Fig. 5 of the main text [1];

Current source 2 -Class VII Current Mirror Current Source given in Fig. 6 of the main text [1];

Current source 3 -Class VI-Symmetrical Widlar Current Source R_E1_=R_E1_=118.7 Ω given in Fig. 6 of the main text [1];

Current source 4 - Class V -Symmetrical Widlar Current Source R_E1_=R_E1_=148 Ω given in Fig. 6 of the main text [1];

Current source 5 -Class IV -Symmetrical Widlar Current Source R_E1_=R_E1_=337 Ω given in Fig. 6 of the main text [1];

Current source 6 -Class III - Widlar Current Source R_E1_=0 Ω,R_E1_=118 Ω given in Fig. 6 of the main text [1];

Current source 7 -Class II - Widlar Current Source R_E1_=0 Ω, R_E1_=148 Ω given in Fig. 6 of the main text [1];

Current source 8 -Class I - Widlar Current Source R_E1_=0 Ω,R_E1_=337 Ω given in Fig. 6 of the main text [1];

source; All together eight current sources are studied by the professional setup.

**Table 6:** Q point of output impedance measurement for a given current source.

Class	I_C1_(A)	I_C2_(A)	V_CEQ_(V)	R_0_(Ω)
Viii	N.A.	992μ	5.019	91.867 k
Vii	1.2 m	1.056 m	4.96	N.A.
Vi	993.4 μ	958.58 μ	4.9	747.8 k
V	1.0577 m	1.0154 m	4.8	838.85 k
IV	918.9 μ	890.16 μ	4.8	1.49 M
III	10.22 m	549.22 μ	5.38	1.127 M
II	10.22 m	549.22 μ	5.36	1.1325 M
I	20.37 m	352.74 μ	5.53	2.26 M

**Table 7:** Hybrid-pi model parameters and T-model parameters (r_02_(kΩ), r_μ2_(MΩ),β_02_, α_02_) at a given Q-point for each class of Current Source.

Class	r_02_(kΩ)	r_μ2_(MΩ)	β_02_	α_02_
VIII	91.1	7.9	71.6	0.986
VII	84.97	7.5	72.5	0.986
VI	93.28	8.02	71	0.986
V	87.12	7.65	72.5	0.986
IV	99.22	8.47	70.5	0.986
III	171.07	13.47	65	0.985
II	170.9	13.45	65	0.985
I	270.6	19.66	60	0.984

**Table 7A:** Hybrid-pi model parameters and T-model parameters(r_x1_(Ω),r_x1_(Ω), r_e1_(Ω), r_e2_(Ω), r_π2_(kΩ), g_m2_(mS) at a given Q-point for each class of Current Source derived from the analytic relations given in the main text.

Class	r_x1_(Ω)	r_x2_(Ω)	r_e1_(Ω)	r_e2_(Ω)	r_π2_(kΩ)	g_m2_(mS)
VIII	N.A.	375	N.A.	26.21	1.88	28.15
VII	375	375	21.49	24.62	1.79	40.62
VI	375	375	26.17	27.12	1.93	38.86
V	375	375	24.6	25.61	1.86	39.04
IV	375	375	28.3	29.21	2.06	34.24
III	286	1k	2.5	47.31	3.08	21.14
II	286	1k	2.5	47.34	3.08	21.12
I	286	1k	1.28	73.71	4.42	13.57

**Table 8:** Comparative study of the analytical values of output impedances obtained for the eight classes of current sources along side the experimentally measured values.

Class	R_S_(Ω)=R_E1_+r_e1_+r_x1_	A(mS)=1/(R_S_+r_x2_)	Z(mS)=1/R_E2_	R_0_CON_ (MΩ)	R_0_UNIV_ (MΩ)	R_0_T-Mod_ (MΩ)	R_0_EXP_ (Ω)
VIII	2.26 M	0	Infinity	0.05	0.09	0.11	92 k
VII	396.5	1.296	Infinity	0.07	0.2	0.33	NA
VI	523.19	1.1133	8.197	0.28	0.8	1.24	747.8 k
V	549.6	1.0815	6.67	0.3	0.84	1.31	838.9 k
IV	730.29	0.905	3.058	0.6	1.44	2.15	1.5 M
III	288.54	0.776	8.197	0.37	1.06	1.74	1.127 M
II	288.54	0.776	6.67	0.4	1.12	1.96	1.13 M
I	251.27	0.497	3.058	0.8	2.23	3.53	2.23 M

Class VII is current mirror. Without matching transistors, Current Mirror cannot be synthesized. Hence the result in this case has been rejected.

Current source 1 -Class VIII-CE BJT current source with constant base current drive given in Fig. 5 of the main text [1];

Current source 2 -Class VII Current Mirror Current Source given in Fig. 6 of the main text [1];

Current source 3 -Class VI-Symmetrical Widlar Current Source R_E1_=R_E1_=118.7 Ω given in Fig. 6 of the main text [1];

Current source 4 - Class V -Symmetrical Widlar Current Source R_E1_=R_E1_=148 Ω given in Fig. 6 of the main text [1];

Current source 5 -Class IV -Symmetrical Widlar Current Source R_E1_=R_E1_=337 Ω given in Fig. 6 of the main text [1];

Current source 6 -Class III - Widlar Current Source R_E1_=0 Ω,R_E1_=118 Ω given in Fig. 6 of the main text [1];

Current source 7 -Class II - Widlar Current Source R_E1_=0 Ω, R_E1_=148 Ω given in Fig. 6 of the main text [1];

Current source 8 -Class I - Widlar Current Source R_E1_=0 Ω,R_E1_=337 Ω given in Fig. 6 of the main text [1];

source; All together eight current sources are studied by the professional setup.

**Table 9:** Voltage gain of the differential amplifier with an active load under experimental condition, under Universal model analysis, under Universal model simulation, and under conventional model simulation.

N	R_S_(Ω)	Exp.Gain(−)ve	Analy. Gain Univ(−)ve.	Sim. Gain Uni.(−)ve	Sim.Gain Con.(−)ve
169	282	2,833	3,773	3,773	908
149	405	2,713	2,843	2,843	785
132	461	2,579	2,556	2,556	739
118	494	2,524	2,412	2,412	715
101	507	2,359	2,360	2,360	705
85	495	2,403	2,412	2,412	715
67	449	2,540	2,612	2,612	749
48	336	2,836	3,299	3,299	850
29	249	3,081	4,137	4,137	948
6	86	2,955	7,889	7,889	1211

**Fig. 7:**
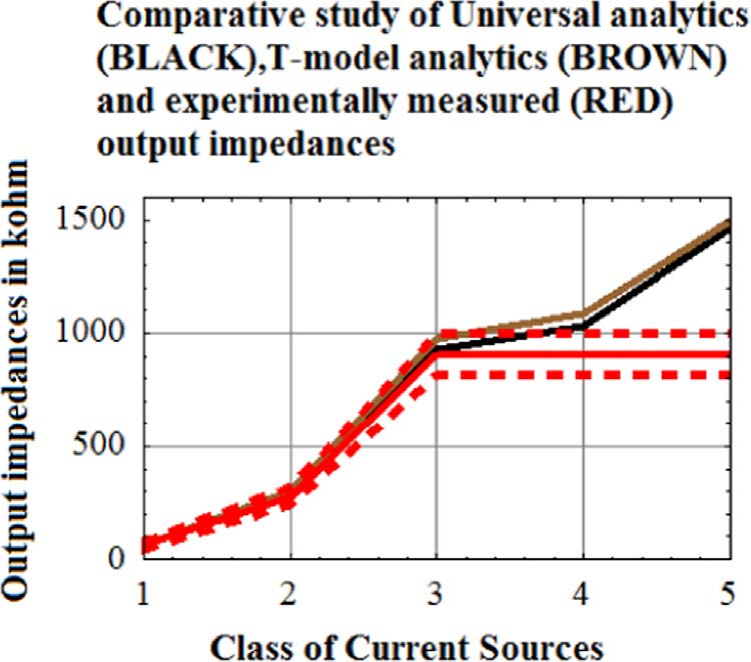
Five (5) classes of current sources are theoretically analyzed using Universal Hybrid-pi model and plotted in BLACK, using T-model and plotted in BROWN and experimentally measured output impedance in kilo-ohm are plotted in RED with dashed lines showing the uncertainty in experimental measurement. (For interpretation of the references to color in this figure, the reader is referred to the web version of this article).

Laboratory setup experimental results donot give unequivocal validation of Universal hybrid-pi model (Fig. 7).

Red and Blue plot are exactly overlapping except at Current 2 corresponding to Class VII current source. This overlap implies that Universal Hybrid-pi model is being validated experimentally with 95% confidence level. At current source 2 (current mirror current source), physically current source 2 could not be synthesized with discrete transistors hence the experimental result for current source 2 could not be procured. This is why overlap does not occur at current source 2.

**Fig. 8:**
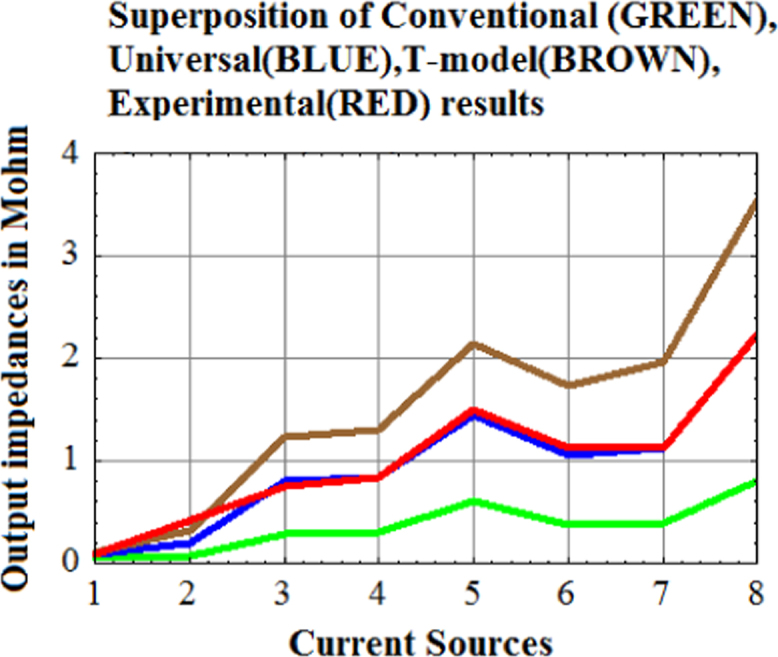
Comparative plot of Conventional Hybrid-pi Model results (GREEN), Universal Hybrid-pi model results (BLUE), T-model results (BROWN) and experimental results (RED). (For interpretation of the references to color in this figure, the reader is referred to the web version of this article).

From Fig. 8, it is also clear that T-model is a systematic over-estimation and conventional model is a systematic under-estimation as is evident from the offsetting of the BROWN curve and offsetting of the GREEN curve with positive error and negative error, respectively.

**Fig. 9:**
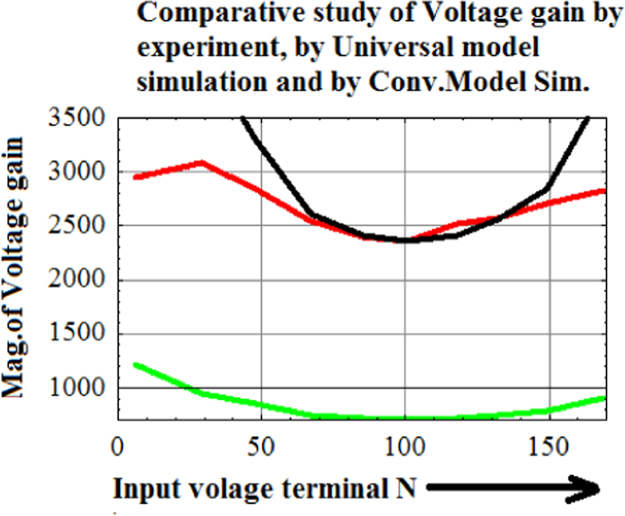
Comparative study of incremental voltage gain of a differential amplifier with Symmetrical Widlar Current source with R_E1_=R_E2_=500 Ω as the active load. Red curve is the experimental plot of incremental voltage gain, Black curve is the predicted gain by Universal Hybrid-pi model, and Green is the predicted gain by conventional hybrid-pi model. (For interpretation of the references to color in this figure, the reader is referred to the web version of this article).

Examination of Fig. 9 clearly establishes Universal Hybrid-pi model as the correct model of CE BJT at low-frequencies. Conventional hybrid-pi model gives 70% underestimation.

## 2 Experimental design, materials, and methods

No experiment is involved in analytical dataset generation hence no material and method for this dataset. The measurement of break-over voltages is done using Tektronix 370 A in curve tracer mode using 2N2219A as the DUT in the circuit under examination. The output current I_C_ is measured and traced with respect to extended V_CE_ until collector current experiences instability and shoots up. This is the break-over voltage. This is repeated for constant current input base current drive (Fig. 5 in the main text of [1]) as well as for constant voltage input base voltage drive. Constant voltage drive at Base corresponds to Current Mirror Current Source. Curve tracing is repeated for symmetrical Widlar and Widlar Configurations of current sources (Fig. 6. in the main text of [1]) .

Details of the use of Tek-370A. This instrument is a Tektronix Curve Tracer for testing devices and circuits. The output curve I_C_vs V_CE_ is traced for a set of base currents. This results in a family of output curves. V_CE_ is extended for zero base current until S-type negative-impedance-curve(NIC) is obtained for zero base current drive. The edge of S-Type NIC is BV_CEO_ (break-over between C and E with Base open) and the vertical to which the S-Type NIC converges asymptotically is V_S_ (sustaining voltage). In current mirror current source, symmetrical Widlar current sources and Widlar Current Sources S-Type NIC does not occur. At the impact-induced instability point (Appendix A-Supplementary data in [1]) the family of curves asymptotically approaches a vertical at BV_CES_, BV_CEX_, and BV_CBO_ for Current Mirror Current Source, Symmetrical Widlar Current Source, and Widlar Current Source, respectively as shown in Fig. 3 of the main text [1].

The laboratory setup for constant current drive current source is given in Fig. 5 of the main Text [1] and the laboratory setup for Current Mirror and Symmetrical Widlar are given in Fig. 6 of the main Text [1]. The incremental voltage across collector resistance is measured using YOKOGAWA Japanese oscilloscope. This incremental voltage divided by the ohmic resistance of the collector resistance gives the incremental collector current drawn by the DUT 2N3055. The incremental voltage across the output node with respect to the ground divided by the incremental collector current gives the incremental output impedance of the given circuit configuration. This is dynamic measurement of incremental output impedance of the given current source at low frequency where the incremental models at low frequency are valid.

In the professional setup ,the measurements have been made over small signal range of 10 mV and are repeatable and reproducible hence small signal condition is approximated and device remains linear . There is <5 percent margin of error. Small signal condition of measurements have been maintained thereby ensuring linearity of the device model and minimizing amplitude distortion. Amplitude distortion leads to reduction in measured values. The circuit is set at the correct quiescent point and incremental voltage is applied in series with the d.c. bias voltage. The incremental voltage across DUT with respect to the ground divided by the incremental current drawn by the DUT gives the incremental output impedance at the output of the circuit under consideration. All measurements are done by SCS 4200 which has nano/pico/femto accuracy. Hence output impedance measurement is of high degree of accuracy. The break-over voltages are also made by SCS 4200.

SCS 4200 gives the bias point of the circuit under consideration.

Incremental model parameters of the DUT being used in the measurement setup are determined from simple analytic relations given below. Base spreading resistances r_x1_ and r_x2_ have been obtained through optimizing the analytic value of the output impedance (given in the main text [1]). The analytic relations for incremental parameters are being restated below:

$$g_{m2}=\frac{I_{C2}}{26\mathrm{mV}};\;r_{e1}=\frac{26\mathrm{mV}}{I_{E1}};\;r_{e2}=\frac{26\mathrm{mV}}{I_{E2}};\;r_{\pi 2}=\beta_{f02}\times \frac{26\mathrm{mV}}{I_{C2}};\beta_{f02}\;\mathrm{and}\;\alpha_{f02}\;\mathrm{are\backslash determined\backslash experimentally}r_{0}=1.076\times \frac{{(0.7586+(V_{\mathrm{CE}}-0.7))}^{0.63}}{0.37}\frac{V}{\mu m}\times \frac{12.9908-{(0.7586+(V_{\mathrm{CE}}-0.7))}^{0.37}}{I_{C}}\frac{\mu m}{A}r_{\mu }=\beta_{f02}\times r_{0}$$

In professional setup,

The measurements have been made over small signal range of 10 mV and are repeatable and reproducible hence there is <5 percent margin of error. Small signal condition have been maintained thereby ensuring linearity and minimizing amplitude distortion. Amplitude distortion leads to suppressed measured values.The circuit is set at the correct quiescent point and incremental voltage is applied in series with the d.c. bias voltage. The incremental voltage across DUT with respect to the ground divided by the incremental current drawn by the DUT gives the static incremental output impedance at the output of the circuit under consideration. The incremental current is determined by noting the increment in collector current. All measurements are done by SCS 4200 which has nano/pico/femto accuracy. Hence output impedance measurement is of high degree of accuracy and is static in nature but it will be identical to low frequency dynamic incremental output impedance.

A differential amplifier using a differential pair constituted of BC548 (matched pair) and a symmetrical Widlar Current source constituted of PNP BC 549 matched transistor pair as an active load gives enhanced gain as predicted by Universal Hybrid-pi model. The minimum voltage source available in the LAB was 10 mV and this was overdriving the very high voltage gain differential amplifier. To reduce the voltage amplitude of the input signal source, a potential divider scheme is used as shown in Fig. 4 in the main text of [1] with 200 in number 10 Ω resistances. *N* is the number of 10 Ω resistance taken for attenuating the input source voltage to an appropriate level so as to drive the circuit within its dynamic range. This keeps the output within the dynamic range, That is we get undistorted sinusoidal output voltage swing for a given input sinusoidal swing with a minimum or no harmonic distortion. Scientific Company (Indore, India) signal generator and Oscilloscope are used for measurement purposes. *Tektronix Digital Storage Oscilloscope.TDS 2012C is used in Math mode to generate the FFT of the signal to test the purity of sinusoidal signal.*
