# Supplementary material for: Dataset of output impedances of current sources by analytical method and by experimental method
Source: Data Brief. 2018 Nov 15;21:2063–74. doi: 10.1016/j.dib.2018.11.050 (PMC6262198; doi:10.1016/j.dib.2018.11.050)
Supplement: Supplementary file 1 — Supplementary material. [file mmc1.docx]

Conflict of interest form

There is no conflict of interest, financial or otherwise, what so ever in this work with any third party.

 Signed by Bijay Kumar Sharma
